# Joint Bayesian Inference Reveals Model Properties Shared between Multiple Experimental Conditions

**DOI:** 10.1371/journal.pone.0091710

**Published:** 2014-04-07

**Authors:** Hannah M. H. Dold, Ingo Fründ

**Affiliations:** 1 AG Modellierung Kognitiver Prozesse, Technische Universität Berlin, Berlin, Germany; 2 Bernstein Center for Computational Neuroscience, Berlin, Germany; University Of Cambridge, United Kingdom

## Abstract

Statistical modeling produces compressed and often more easily interpretable descriptions of experimental data in form of model parameters. When experimental manipulations target selected parameters, it is necessary for their interpretation that other model components remain constant. For example, psychophysicists use dose rate models to describe how behavior changes as a function of a single stimulus variable. The main interest is on shifts of this function induced by experimental manipulation, assuming invariance in other aspects of the function. Combining several experimental conditions in a joint analysis that takes such invariance constraints into account can result in a complex model for which no robust standard procedures are available. We formulate a solution for the joint analysis through repeated applications of standard procedures by allowing an additional assumption. This way, experimental conditions can be analyzed separately such that all conditions are implicitly taken into account. We investigate the validity of the supplementary assumption through simulations. Furthermore, we present a natural way to check whether a joint treatment is appropriate. We illustrate the method for the specific case of the psychometric function; however the procedure applies to other models that encompass multiple experimental conditions.

## Introduction

Many experiments in the quantitative sciences are set up to manipulate a single or a few selected model parameters, assuming that other parameters of the model remain constant across the different experimental conditions. Thus, there are some parameters in the model that are determined by the general experimental setup; there are other parameters in the model that are expected to vary with experimental conditions.

The so-called dose rate model is an important model in many natural sciences. Dose rate models describe the probability that some event occurs as a function of some independent “dose” variable. In psychophysics, for example, dose rate models describe the probability that an observer detects a given pattern as a function of the contrast of the pattern [1]–[5]. Other examples can be found in medicine—the probability of therapeutic success as a function of dose of some medication, or in toxicology—the fraction of test animals that die after application of some toxic substance [6], [7]. To compare sensitivity changes for several patterns, medications or toxic substances, several experimental conditions are measured, their corresponding dose rate models are computed and contrasted.

In the simplest case, all dose rate models can be handled within the framework of generalized linear models [8], [9] which provides numerically efficient ways of estimation and has well established procedures to check for goodness-of-fit. Estimation of generalized linear models is also easy for multiple dependent variables and thus for multiple conditions. For generalized linear models to be applicable, the dependent variable, a probability, must take the lowest plausible value of zero and the highest plausible of one. However, in many of the above examples the lowest plausible value for the dependent variable is actually larger than zero, though; and the highest plausible value for the dependent variable is smaller than one. In psychophysics, observers might have a certain probability to guess correctly, even if the stimulus was much too weak to be detected by the eye [2]. In medicine, there might be spontaneous remissions, and in toxicology, some of the test animals might be resistant to the toxic substance. In these cases, the asymptotic levels (spontaneous remissions, guesses,...) need to be estimated, too. If they are not estimated, this might result in estimation biases for the actual parameters of the dose response curve [3]. However, including these parameters renders the likelihood function of the model non-concave and in many cases multimodal—a fact that seriously complicates model estimation. Consequently, software that can be used to perform inference in such models [3] typically employs methods for global optimization such as grid searches or Monte Carlo procedures.

A dose rate model with asymptotic levels is designed for and works well with the estimation of a single condition. An extension of the model to encompass multiple conditions bears a few difficulties. First of all, the parameter space that would need to be searched by these global optimization routines grows exponentially with the number of added conditions. Thereby, numerical stability and efficiency might be sacrificed. Furthermore, goodness-of-fit [10] as well as other routines, e.g. determining influential observations in the data set [11], becomes more difficult to judge. From a practical point of view it can be said that fitting a dose rate model for individual cases is a standard routine for a psychophysicist or toxicologist. A deviation from standards needs always a double thought since the standard was tailored to the problem by the needs of the field. Our goal is therefore to handle several data sets simultaneously but by extending the common routines and keeping their advantages, not by changing to a more powerful methodology.

Instead of fitting all conditions in a common model [12], we suggest an alternative approach that still models each condition individually as a dose rate model. Yet, information from all other conditions is incorporated into the inference. Bayesian statistics allows for a very natural way to include external information into the inference process. In Bayesian statistics, the external information is typically incorporated in the form of a “prior” probability distribution because it describes all the information available to an experimenter before he or she has seen the data that are actually analyzed. Here we propose a method to derive prior distributions that integrate information from other experimental conditions and pose an implicit constraint to force a desired parameter to be equal across conditions. An applied example of this procedure is provided in the section “Example from perceptual psychology: the psychometric function” and further elaborated in section “Another example and statistical tests”.

## Method

### Separate sampling for joint inference

**Figure 1:**
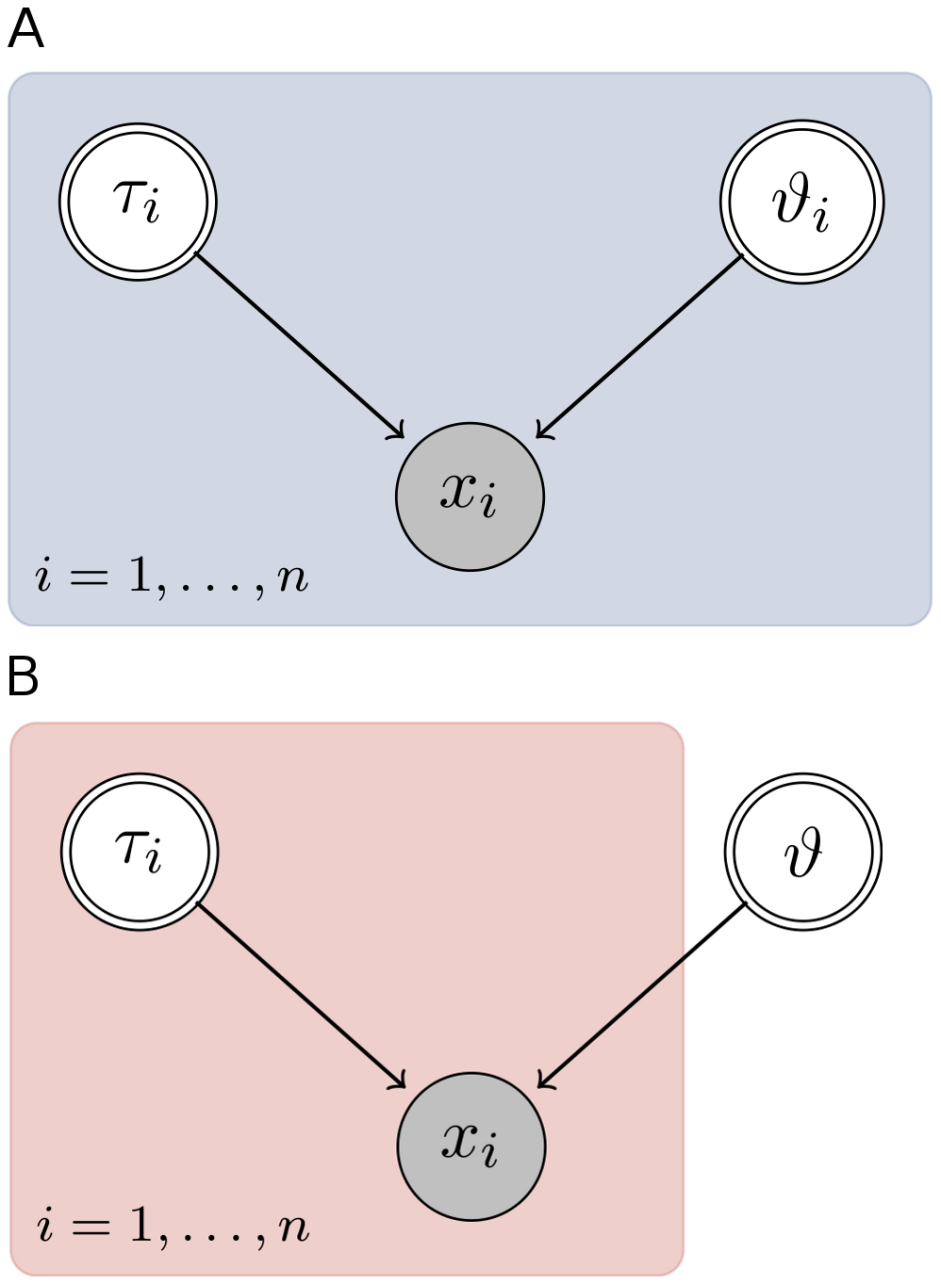
Graphical models illustrating the isolated models and the joint model approach. A graphical model illustrates the dependencies between the variables of a model. The plates in the background of both graphical models group variables according to condition *i*. In panel A, the observed variable 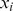, the data from condition *i*, depends on the random variables 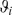 and 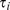, each being characteristic for condition *i*. The only difference in the second panel is that the random variable 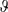 is outside the background plate, which means the variable does not depend on condition *i*—it is shared across conditions.

To explain the ideas behind joint inference, we imagine that *n* data sets were collected experimentally, one data set per experimental condition. Each data set, 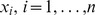, can be described by the same model *M* with parameter vector 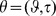, but the specific parameter values of *M* might differ with condition *i*. Note that 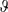 and 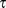 can be scalar as well as multidimensional. The standard analysis treats each data set individually; each condition is analyzed separately. We will refer to this collection of fitted models as the *isolated models*. The graphical model for the isolated models *M* is depicted in Figure 1A. Each node represents a factor in the joint distribution of model variables. The filled node stands for the observed variables, 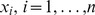 and represent the data sets collected for *n* different conditions. The random variables 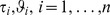 are drawn as double circles. The blue plate in the background groups variables that belong to the same condition *i*. We assume here, that the structure of the submodels for each condition is known and only the associated parameters need to be identified from the data. For the case in which the model structure itself is to be inferred from the data, see [13].

The goal of joint inference however is to fit all conditions simultaneously, because the experimenter suspected that one parameter, say 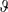, is shared across conditions. The graphical model for the joint analysis is shown in Figure 1B and we will refer to this model as the joint model. Such a situation can arise, for example, if the system described by the model has some parameters that are dependent on the state of the system, here 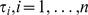, and some that are state independent, 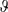. The difference between graphical model of the isolated and joint models is that 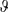 does not depend on *i*. In the following blue colors are used for results from isolated inference and red colors for results from joint inference. We will show next how the computation of the isolated models in a first step can serve to fit the joint model. The method is illustrated with 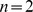 data sets.

The main assumption for the joint inference procedure is that the parameters in 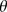 are a posteriori independent. The assumption's appropriateness is further investigated in the section entitled “Evaluation of the method”. For parameter posterior distributions of the isolated model 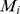 follows that
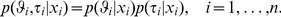
(1)


Further, we exploit the fact that an independent distribution can be represented through its marginals. Given the assumed a posteriori independence of the model parameters, we can write the joint parameter in the joint model via the marginal:
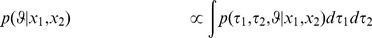
(2)

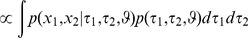
(3)

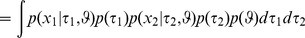
(4)

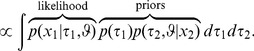
(5)


Here, we used Bayes Theorem, the a priori independence of model parameters, and finally reorganized the terms to arrive at an expression that we will use next to sample from the posterior. This expression suggests a reinterpretation in the form of likelihood and prior terms: The “likelihood” only contains the first data set 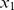. The second data set 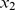 appears in one of the prior terms. If the joint model is a correct description of the data, then the shared parameter 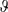 equals the parameters of the isolated models and 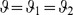. The previous equation can therefore be rewritten by replacing 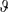 by 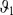 and 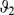 to arrive at,
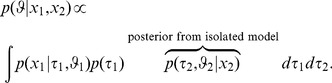
(6)


The term that works as a prior for 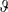 resembles the posterior of the isolated model applied to the second data set (equation (1)). As a result the posterior of 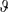 in the joint model that is based on all data sets simultaneously can be estimated in a two step procedure. The first step is to determine the posterior of the isolated model on the second data set 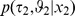 and determine its marginal 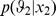 from (1). In the second step we estimate the parameters of the isolated model from the first data set 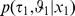 using the marginal 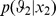 as a prior for 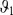. Thus, it is possible to reduce the formulation of the joint model to a sequence of isolated models.

The terms in equation (4) may be ordered differently. This way, we can apply the isolated models in a different sequence.
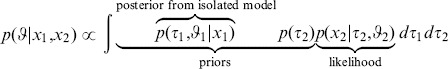
(7)


Theoretically, the order should not matter and the marginal posterior distributions computed with different orders are equivalent. This can be used as a sanity check. If the full model is an adequate description of the data and 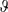 is shared across conditions, the marginal posteriors of the parameters obtained in different orders overlap. Caution must be taken in applications where the posterior distributions are approximated. If the approximation is not stable or too coarse, the order in which the data sets are analyzed will impact the final result. For example, we approximated the posterior distributions in the remainder of this study using Monte Carlo samples. If the number of Monte Carlo samples is too low, joint posteriors obtained with different orders may be different. In that case, it is necessary to generate more Monte Carlo samples, or otherwise improve the approximation to the posterior distribution.

**Figure 2:**
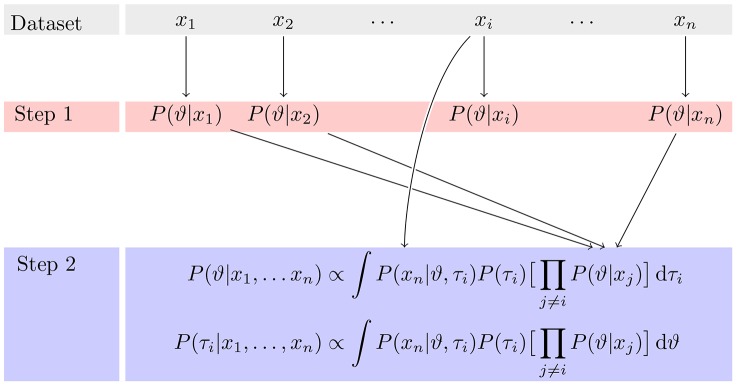
Illustration of the procedure for 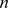 data sets. The second step is show here for the general condition 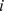. The marginal posterior distribution of the shared parameter 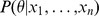 does not depend on 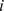. Yet, for the non-shared parameters 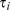, the marginal posterior distribution depends on 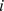.

The approach is extendable to *n* data sets, which we will summarize briefly (see also figure 2): In a first step the parameter posterior distributions for each of the *n* conditions are determined in isolation. The marginal posterior distributions of the shared parameter from 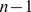 conditions are multiplied and this product is the prior for a second round of inference on the 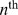 condition. In contrast to the first step, the second step introduces information from all other conditions into the inference procedure. Thus, after this second step, the marginal posterior distributions of the shared parameter are the same across all conditions. This way, the second step of inference implicitly performs inference on all conditions simultaneously. In the next section, we will illustrate the strategy from the previous section for a concrete example from perceptual psychology.

### Ethics statement

The following example as well as the example in the section entitled “Another example and statistical tests” are reanalyses of data recorded by Wichmann [14]. These data have been collected after obtaining the informed consent of the tested observer. Given that in the original study, the experimenter collected data on himself, a written statement of consent was not deemed necessary. At the time when the data were collected in the psychology department of the University of Oxford, there was a general waiver that provided general approval for psychophysical experiments. The procedures were in accordance with the declaration of Helsinki.

## Results and Examples

### Example from perceptual psychology: the psychometric function

The psychometric function relates the performance of an observer to the intensity of a stimulus. Here, intensity can be the sound pressure of an auditory tone or the contrast of a visual stimulus. Performance is typically expressed in terms of the probability that the observer correctly detects a predefined target stimulus.

We analyze psychometric function data from a single observer in an experiment by Wichmann [14]: The observer performed a two alternatives forced choice task in which he had to monitor two time intervals. Each interval lasted 79 ms. In one of these two intervals, a low contrast sinusoidal target grating with a spatial frequency of 8.37 degree visual angle was presented. The observers task was to identify which one of these two intervals contained the target grating. At each contrast level of the target grating, either 40 or 50 responses were collected. Performance was measured as the fraction of trials in which the observer correctly identified the interval that contained the target grating. We analyze data that were collected in two different experimental conditions: First, a “masking” grating of low contrast (Michelson contrast of 1.6%) was presented in both intervals. The mask was in phase with the target grating such that the task was essentially to identify the interval in which the grating had higher contrast. We will refer to this condition as the “low contrast mask” condition and present corresponding data in a light color scheme. In the second condition, the mask had a high contrast (Michelson contrast of 51.2%). We will refer to this condition as the “high contrast mask” condition and use a darker color scheme to show data and results. All experimental data used in this study are available from http://www.ingofruend.net/jointbayes.html.

To model these data, the responses were assumed to be binomially distributed with a probability of success given by [3], [15]

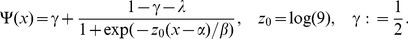
(8)This model has three free parameters 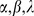. The parameter 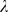 describes the upper asymptote of the model and is treated as a nuisance parameter. Although 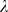 is usually not of scientific interest, omitting 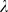 from the model introduces potential estimation bias in the other parameters [3]. The remaining two parameters 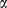 and 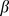 are psychologically interesting: 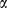 is the stimulus intensity at which the psychometric function is halfway between the lower asymptote, 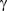, and the upper asymptote 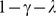. Thus, 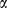 is often reported as the *threshold* and 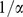 can be considered a measure of sensitivity. The other parameter of interest is 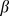, which is proportional to the slope of the psychometric function at a contrast of 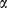: If 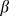 is large, the psychometric function is very shallow, if 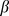 is small, the psychometric function is very steep. By incorporating the constant 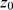 into the equation, 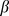 gives the range of stimulus intensities on which the psychometric function rises from a performance level 10% above the lower asymptote to 10% below the upper asymptote. Thus, this parameter is the width of the range of contrasts over which the observer's performance is sensitive to changes in the stimulus contrast. We estimated the parameter posterior distributions by a sampling-importance-resampling procedure [16], [17]. Sampling-importance-resampling uses an arbitrary distribution to generate a number of proposal samples. Each proposal sample is then assigned a so-called importance weight, which quantifies how important that sample is to represent the target distribution. Finally samples are drawn from the proposals with probabilities proportional to their importance weights. We proposed 25 000 samples to arrive at 2 000 final samples here. The prior distributions in the isolated inference or the non-shared parameters in the joint inference procedure were conjugate priors to the posteriors typically observed in experiments.

Wichmann [14] reports no strong changes in slope for different masking contrasts. This means that the slope could be modeled as a shared parameter, and we illustrate the method based on this data in the remainder of this section. In a later section called “Another example and statistical tests”, we will investigate a second scenario in which the assumption of parameter equaility is not valid, which means that the data do not originate from the same distribution. Here, we will contrast fits and posterior distributions obtained with the standard isolated inference procedure and the newly proposed joint inference procedure. Both procedures were applied to exactly the same data sets.

**Figure 3:**
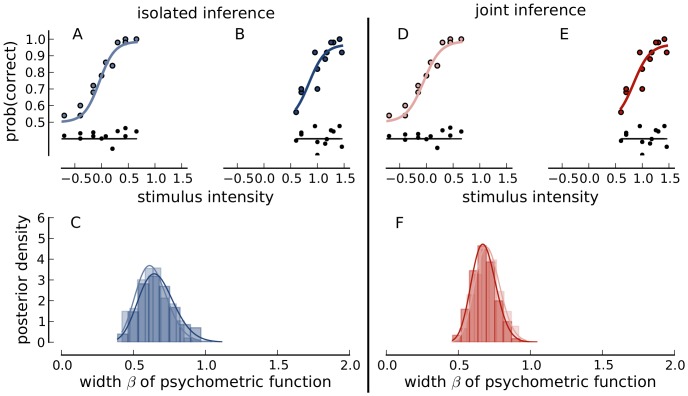
The procedure applied. Panel A, B, and C use the isolated inference procedure, Panel D, E, and F the joint inference procedure. Psychometric functions, shown as solid lines, were fitted to a dataset using a low contrast mask (Panel A) and a high contrast mask (Panel B). Deviance residuals are shown in black below data and fit. Panel C shows the marginal posterior distributions of parameter 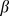 from both data sets in the corresponding colors to the fits above. Panel D and E are equivalent to Panel A and B except that the joint procedure was applied, which uses the marginals from Panel C as prior distributions. The marginal posterior distributions of 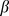 are shown in Panel F.

Figure 3 illustrates the results of the analysis. We first discuss the results when each data set was fitted independently. Figure 3A and B display the data (dots) as well as the Bayesian posterior mean estimate of the corresponding psychometric function. The functions provide visually good fits although the high contrast mask (Figure 3B) data scatter slightly more around the fitted function than in the low contrast mask (Figure 3A) condition. The deviance residuals plotted below the curve capture this observation well.

Figure 3C shows marginal histograms of samples from the posterior. We tried to summarize the samples by fitting them with a parametric model. The solid lines in the second row are maximum likelihood fits of Gamma distributions to the samples from the marginal posterior distribution. We observe that the histogram for the “low contrast mask” condition (Figure 3C light blue) is very similar to the histogram for the “high contrast mask” condition (Figure 3C dark blue). Furthermore, the histograms are very well approximated by fitted Gamma distributions. We took these fitted Gamma distributions as parametric summaries of the posterior samples.

With this prerequisite, a joint fit of the psychometric functions in the two masking conditions might succeed. Indeed after applying the procedure presented in the section “Separate sampling for joint inference”, the fits remain very good. The joint mean a posteriori fits in Figure 3D and E fit the data nearly as well as for separate inference. Note here, that even the residual plots in the bottom part of Figure 3D and E are very similar to those for isolated fits (Figure 3A and B). Also, the a posteriori histograms in Figure 3F are highly overlapping. It should however be noted that neither the histograms nor the fitted parametric summaries are exactly the same. However, once the experimenter has decided to accept the joint inference to provide valid results, the posterior samples stem from the same distribution, the joint posterior distribution. Thus, by accepting the joint inference, the experimenter assumes that differences between histograms only reflect the sampling errors during posterior sampling.

### Evaluation of the method

The previous section illustrated the method through an example from perceptual psychology. In this section we will use the same model as in the example to evaluate the method with respect to two questions that concern the general applicability. First, by definition our method requires that the posterior parameter distributions after the first sampling round can be represented by its marginal distributions without loss of information. This is only true if the parameters are a posterior independent. Here, we investigate how crucial this independence assumption is. Second, we study the success of the method. Success means to achieve an overlap between the marginal posteriors without impairing deviance. We address both questions by simulating data from functions with known parameters and applying the method on pairs of these synthetic data sets.

The synthetic data sets used in this section were all generated from the same underlying psychometric function 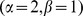. That means, by design the joint inference procedure is legitimate to use. The data sets differ with respect to their sampling scheme—the intensities at which the psychometric function is evaluated— and the number of responses per stimulus intensity (trials). Both were chosen randomly for each data set. The number of trials per intensity block ranged between 20 and 200. Six intensity levels were chosen randomly to sample the psychometric function. It was assured that they covered certain intervals in the asymptotes and rising part of the psychometric function. Thereby, the data sets differ in the amounts of correlation between the parameters. We observed that properly sampled psychometric functions [3] exhibit only minor correlations between parameters. Thus, the assumption will typically be justified in practice. We quantified how well the procedure works dependent on the correlation between 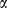 and 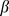. This seems sufficient, since 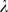 is only a nuisance parameter. For the quantification we chose two statistics, one that captures goodness-of-fit and one that captures the overlap between the posterior distributions of the joint parameter. The procedure works well, if the goodness-of-fit is nearly the same between isolated and joint fits, and if the overlap between the posterior distributions increases.

**Figure 4:**
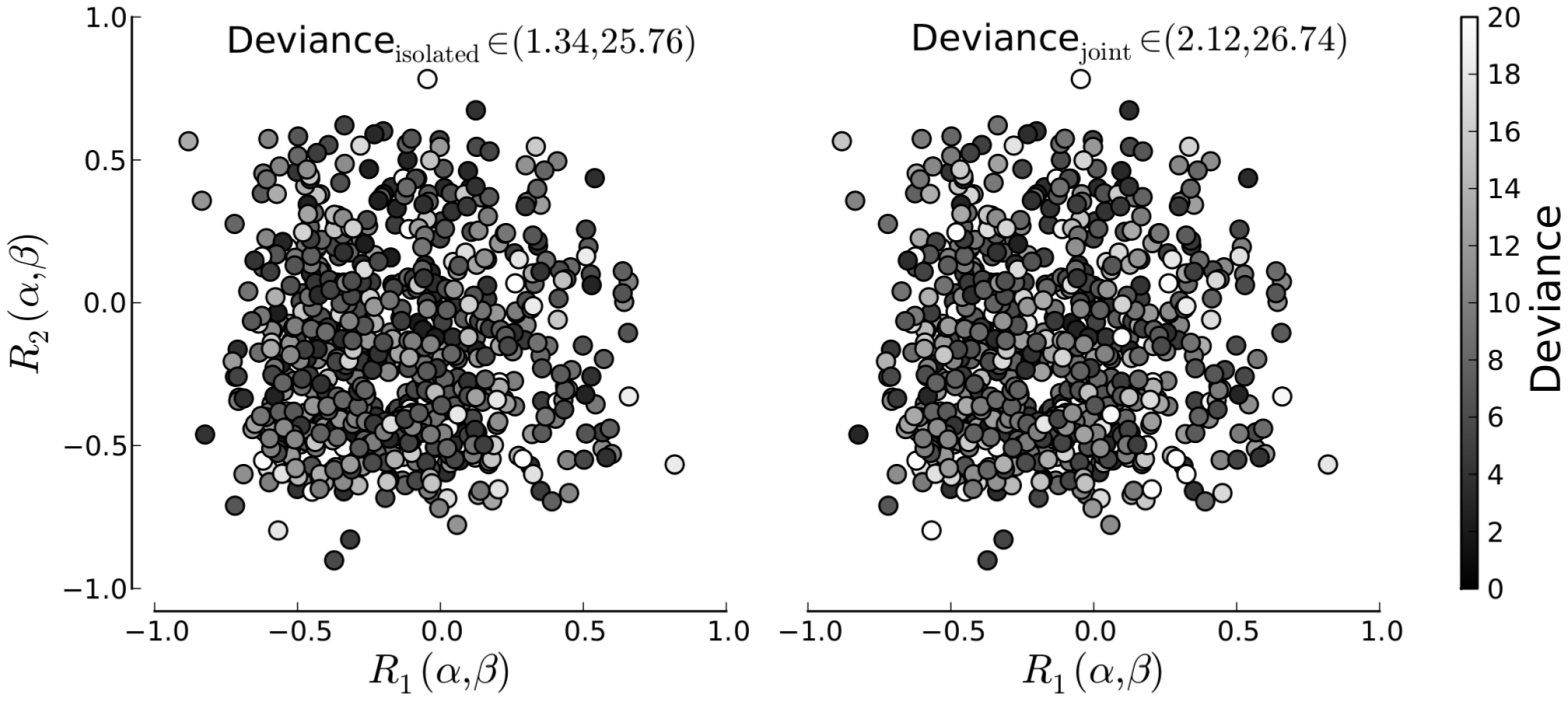
Deviance as a function of parameter correlations for the isolated models(left) and joint model(right). The color of each data point corresponds to the combined deviance obtained through psychometric function fits to two artificially generated data sets with the same generating parameters. The cardinal axis denote the correlation between the generating parameters 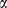 and 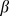 in the first and second data set.

Goodness-of-fit of a single condition was quantified by deviance:
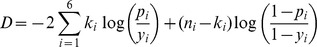
with the number of intensity levels, *i*, the number of trials, *n*, the number of correct responses, *k*, the model prediction, *p*, and the observed performance, 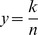. To compare the fit of the isolated models with the fit of the full model, the deviance of the *n* model components are summed: 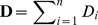. Figure 4 shows goodness-of-fit as a function of the correlation between the two parameters estimated for two data sets. The first panel presents deviance sum 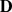 of both data sets fitted in isolation. 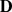 is plotted against the correlation value 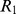 of 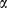 and 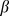 in the first data set and the correlation 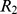 in the second data set. The better the fit, the smaller deviance and the darker the color. The second panel presents 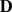 for the same data sets, but after the joint fitting procedure. The color pattern of Figure 4 shows that, first, there is no trend with correlation and, second, the deviance pattern is very similar for isolated and joint inference.

**Figure 5:**
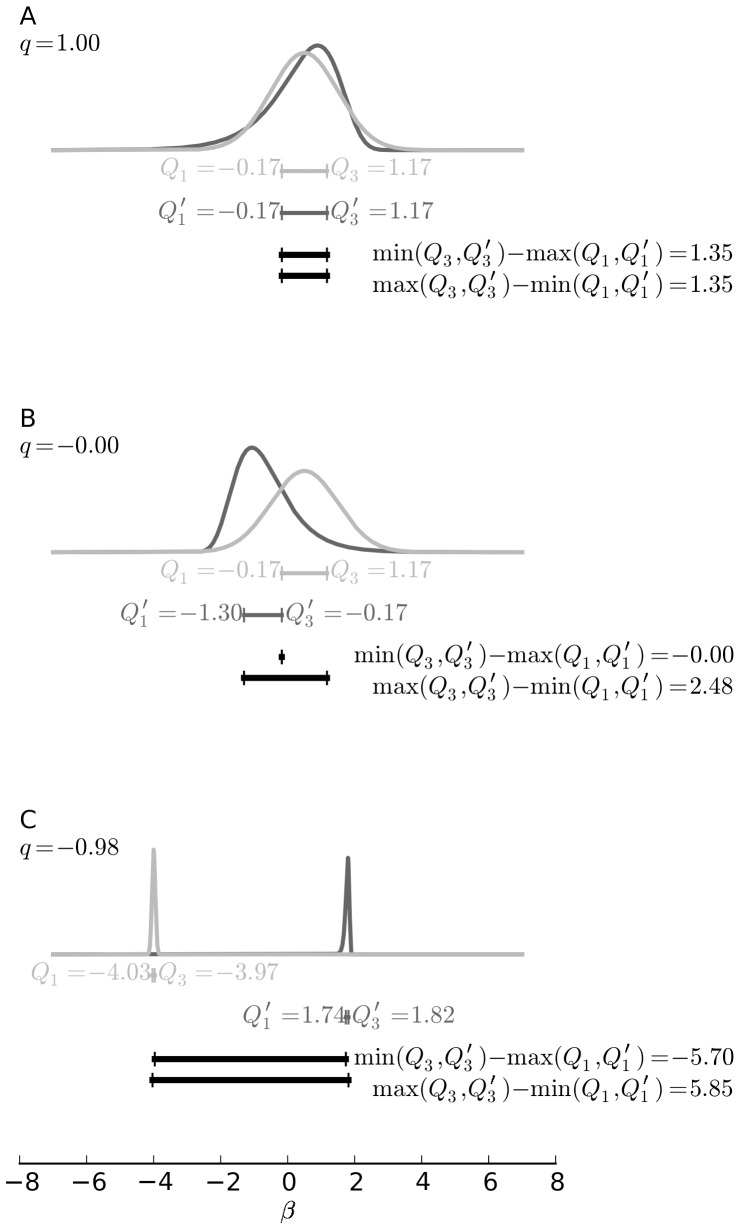
Overlap statistic explained. Panel A shows two distributions with the maximal value of overlap 1. The distributions themselves are not exactly equal, but their first and third quartiles, 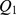 and 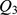, are. The intermediate steps to compute the overlap as denoted in Equation 9 are shown below the distributions. Panel B shows an example of distributions, quartiles and intermediate results that result in a overlap of 0. In this case the interquartile range of one distributions ends where the second starts. Panel C contains distant distributions with their quartiles and results. Here the width of the interquartile range is negligible compared to the distance between the distributions. The resulting overlap statistic is −1.

To quantify the overlap between two distributions, we compute a statistic based on the first and third quartile of the distributions. We prefer this statistic over other options such as KL-divergence, because it is a simple, robust measure with respect to the mass of the sample distribution where the exact shape of the distributions and the tails are not that important. Figure 5 illustrates the statistic. Let the quartiles be 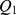 and 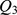 for one distribution and 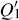 and 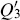 for the second distribution. The overlap is computed by:
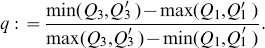
(9)


This means, that if the distributions are very similar and the quartiles fall on the same values, then the overlap is 1 (Figure 5A). If the interquartile ranges overlap partly, the result is positive. The overlap is 0, if one interquartile range starts where the other ends (Figure 5A) and grows negative with the limit of 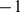 if the distributions diverge (see Figure 5C for an extreme example).

**Figure 6:**
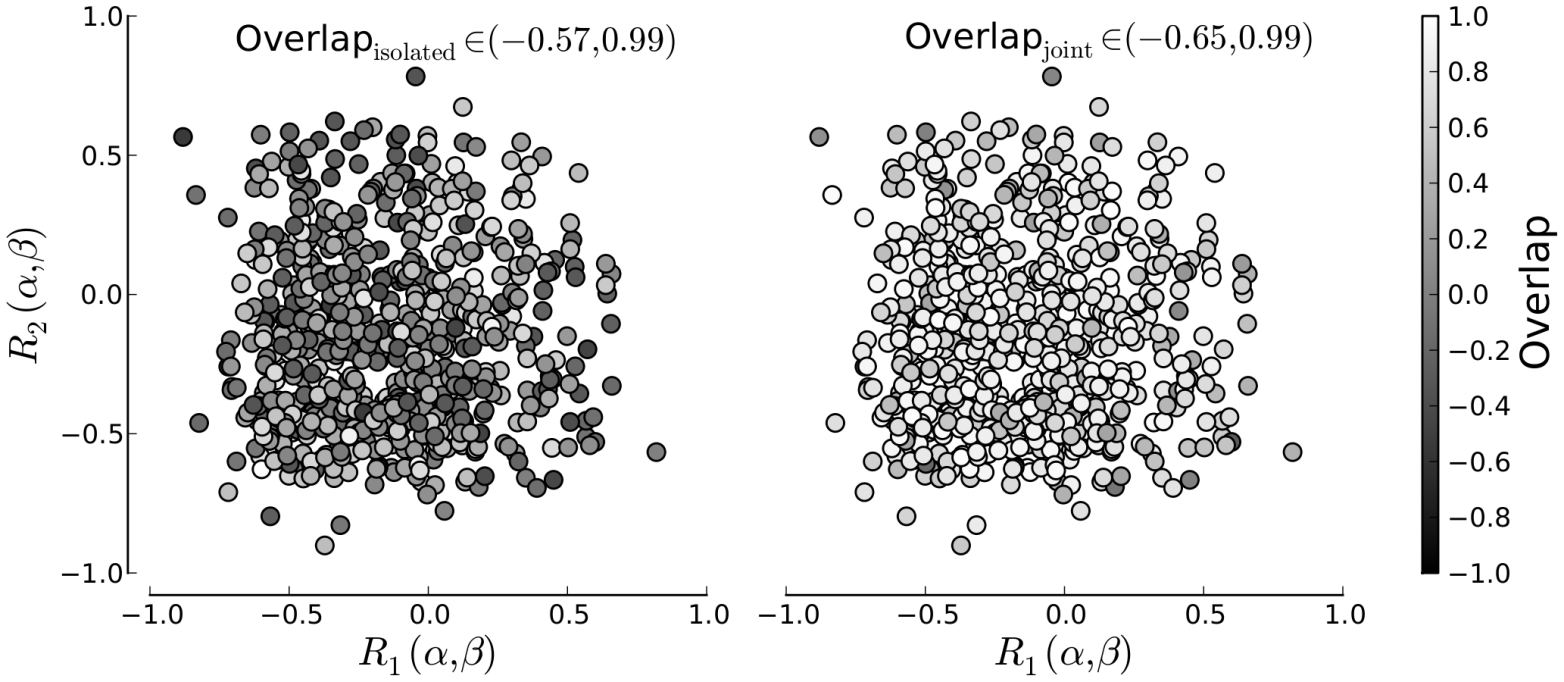
Overlap as a function of parameter correlations for the isolated models(left) and joint model(right). The color of each data point corresponds to the overlap of the posterior marginal distributions of parameter 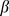. Despite the fact that the generating parameters of the data sets were the same, the inferred parameter distributions can show rather low overlap in the isolated model approach. The cardinal axis denote the correlation between the generating parameters in the first and second data set.

Figure 6 presents the overlap *q* between the posterior distribution of the width parameter 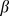 as a function of the correlation structure of the data sets. Again the results are shown for both, isolated and joint fits. The lighter the color, the greater the overlap. As for deviance the figure shows no trend of the overlap dependent on the correlation between 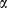 and 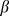. We would like to point out, that the initial overlap between the marginal posterior distributions is rather low, even if the generating functions of the data sets were the same in this example. This is due to the rather large variance of binomially distributed data, especially for small data sets. With joint fitting the overlap increases strongly and results in mainly positive indices.

**Figure 7:**
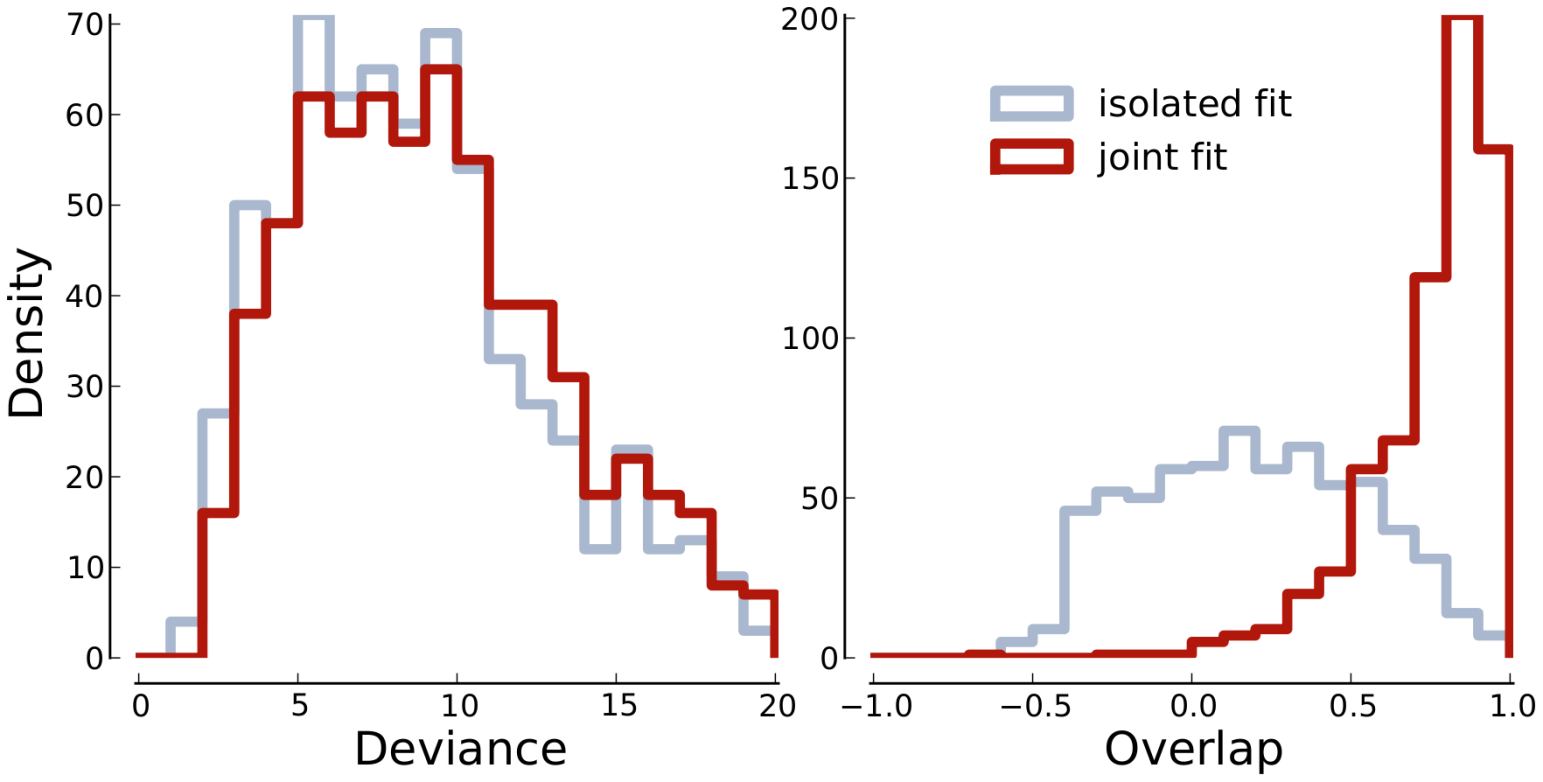
Histograms of the deviance and overlap data shown in Figure 4 and Figure 6. The dark histograms corresponds to the joint and the light histograms to the isolated fits.

The simulations show that neither deviance nor overlap are sensitive to the assumption of parameter independence. This allows us to summarize the results across correlation and present them as histograms in Figure 7. The first panel shows histograms of deviance as obtained with isolated and joint fitting in blue and red, respectively. The second panel shows histograms of overlap also for isolated and joint fitting using the same color code. The deviance histograms are rather similar while the overlap histogram shifts clearly towards larger values for the joint fit. In combination the results presented so far imply, that the method is robust in the case of data from the same underlying function.

### Another example and statistical tests

For the example presented above, it was reasonable that the parameter, on which the method was applied, did not differ between data sets collected in different experimental conditions. For the simulated data it was even guaranteed by design. We have shown that in this case the joint fitting procedure resulted in model fits that were as good as the benchmarks obtained in isolated fits with the additional gain of approximate equality of one of the parameter posterior distributions. Clearly, in any true experimental setup, data from different conditions will not be from the same distribution. In this case, it may still be desirable and parsimonious to treat some parameters of the model *as if* they had the same distribution. Thus, in these cases, there is a trade-off between achieving approximate equality of one (or more) marginal distributions of the posterior on the one hand and maintaining a good fit on the other hand. This section presents data and statistical analysis of a scenario in which parameter equality is not guaranteed and we show how this situation can be handled.

Wichmann [14] reports no strong changes in the width of the psychometric function for different masking contrasts which is consistent with our results in the previous sections. However, if observers had to discriminate a target grating from a homogeneous background—the “no mask” condition—he reports a decrease in width.

Again we analyze data that were collected in two different experimental conditions: We reuse the data previously called the “low contrast mask” condition (light color) and add the “no mask” condition (dark color).

**Figure 8:**
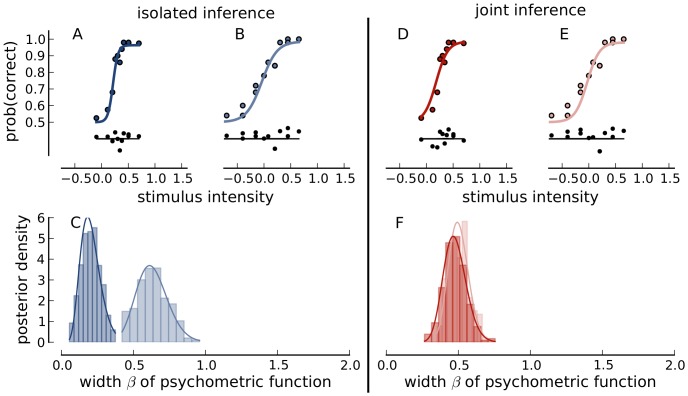
The procedure applied to different data sets. This figure is constructed equivalently to Figure 3, but different data sets are used. In Panel A and D an experimental condition without a contrast mask is shown. Panel B and E contain the same data sets as Figure 3 A and D. In this example, the marginal posterior distributions from the isolated inference procedure result in markedly different parameter posterior distributions (Panel C) which are forced to overlap through joint inference (Panel F).

Indeed, the psychometric function in the no mask condition (Figure 8A) is slightly steeper than in the two masking conditions (for example “low contrast mask condition” in Figure 8B). Also the histograms (Figure 8C) are quite different for the no mask condition as compared to the low contrast mask conditions. In general, 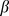 tends to be lower in the “no mask” condition. This reflects the previous result by Wichmann [14] on the same data that psychometric function slopes were markedly different if a mask was present or not.

It is clear that these two marginal distributions are considerably different. There is only little overlap between these two distributions. Nonetheless, we can use the method presented in the section “Separate sampling for joint inference” to force the two posteriors to be (approximately) equal. The psychometric functions corresponding to the resulting mean a posteriori estimates are shown in Figure 8D and E and the respective a posteriori distributions in Figure 8F. The procedure results in posteriors that are closer together. However, the fit quality is worse than for the two separate fits: In the no mask condition (Figure 8D), the fitted function is consistently above the recorded data points at low signal contrast, while in the low contrast mask condition the fitted function is consistently below the recorded data points (Figure 8E).

To decide whether the joint model or the isolated models provide a better description of the data, we use model selection by treating the model itself as another parameter and determining the marginal posterior distribution of this parameter. With a flat prior, this is equivalent to a decision based on the Bayes factor (see for example [18] for a review and tutorial). To do so, we derived samples for the isolated model as well as the joint model. In the next step, we considered the posterior distribution the joint space of models and parameters (“model” has two possible values “isolated” and “joint”). In Methods S1, we show how the marginal model distribution in this space can be obtained trough Gibbs sampling and that it is even possible to approximate the stationary distribution analytically.

Again artificial data sets were generated to quantify the sensitivity of the model selection approach. In the previous simulations we observed, that large correlations only occurred if the psychometric function were not well constrained by the data points. For example, if no data was collected in the raising part or in one of the asymptotes. Realistic sampling schemes, as one would demand for meaningful experimental data, did not yield large parameter correlations. We took advantage of that observation and selected only data sets with a correlations of less than 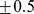. In contrast to the previous simulations, here the simulated data sets could differ with respect to the widths of their generating psychometric functions. The models posterior probabilities obtained with data sets having no difference between the width of the generating functions, then we expect the model posterior probability of the joint model to be at least equal to the model posterior probability of the isolated models. The joint model could even be favored because it is simpler. Simplicity in this context is expressed in the area covered by the prior distributions of all parameters together. If the functions that generated the data sets had truly different slopes, we would like our method to prefer the isolated model. Obviously, it might be impossible to discriminate “equal slopes” from “very similar but not equal slopes” on finite data sets. Thus, if the width difference between the generating psychometric functions of two data sets is sufficiently small, we would like our method to consistently prefer the joint model.

**Figure 9:**
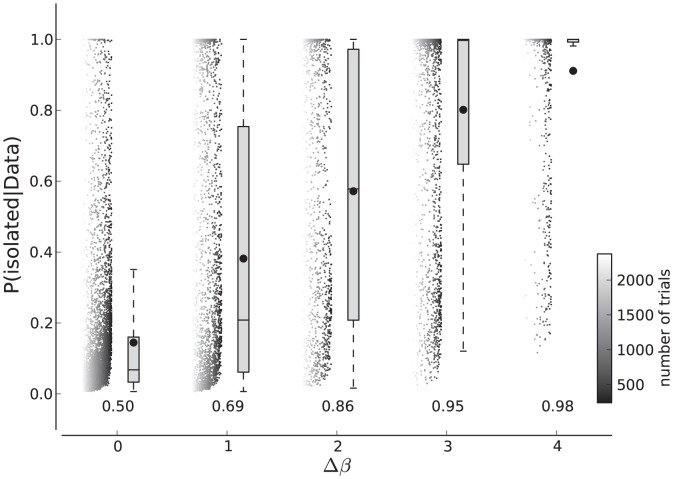
Model selection between isolated and joint model. For each data point in this plot, two artificial data sets with a difference of 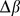 in their generating functions were used. The probability of isolated models being the basis of the data sets, and not a joint model, is shown for differences from 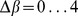. The color of the data points denote the total number of trials in the data set pair. The probabilities of each 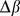 are also summarized in boxplots. The comparison of hits in the condition when 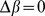 with false alarms in the present condition result in an AUC characteristic shown in the number given for each difference.

Figure 9 shows boxplots and the mean of the isolated models' posterior probability depending on the true width difference between the generating parameter, 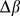. The scattered values are the raw results colored by the number of trials in the data sets. Applied on our simulations we find that the isolated models' posterior probabilities accumulate below values of 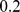 if 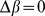. With increasing 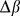 the main support of the box plot and individual results in the scatter shift towards 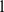. The probability of the isolated models increases with 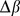 as is expected. We had a closer look on the simulations where the data sets were generated with very different slopes but where the model posterior favored a joint analysis. Many of those data sets contained samples that did not describe the psychometric function well. Either these data sets were lacking samples in the raising part or samples in one of the asymptotes. In a real scientific experiment psychometric functions with this property would not be tolerated and more data would need to be collected. The consequence of such data is that the prior from the other condition faces no conflicting data and a joint fit is feasible. The strong scatter of the model posteriors therefore stems from the limited number of data samples—here six—combined with an unfortunate positioning of intensity values. The shift of the distributions from 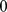 to 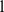 with increasing width is slower than it would be with realistically sampled psychometric functions. Of course, it is important to show that the model comparison works as expected. However, as a scientist one is interested in the separability between the simulations that allow the joint procedure and the simulations that do not. Therefore, we also computed the “area under the curve” (AUC)— a measure for linear separability between two distributions analyzed in a receiver operating characteristic. The values given for each 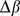 in Figure 9 are the characteristic computed for that 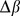 and 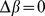. A value of 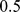 indicates that the distributions are completely overlapping and separability is impossible. A value of 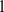 indicates perfectly separable distributions. The AUC increases quickly with 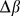. Note that the observed AUC values underestimate the power of the procedure which would be obtained with better defined psychometric functions.

Coming back to the our two examples, here Bayesian model selection as described above gives the following results. In the first example with the low and high contrast mask, the slopes appeared equal. Here, the posterior probability should favor the joint model. Indeed, the posterior probability for the joint model is 0.977 in the first example. In the second example presented at the beginning of this section, one condition did not contain a mask at all. We claimed that the slopes were different in this case. Here, the model selection strongly supports the isolated models with a posterior probability of 0.997 for the isolated models. Therefore, and consistent with the visual inspection of psychometric functions, the joint procedure should be only applied on the masked conditions, but not in the second example with and without a mask condition.

## Discussion

We presented a Bayesian approach to perform joint inference. By joint inference we mean to perform inference on the basis of several data sets simultaneously. The main difference to other procedures is that the data sets are fitted individually by taking all available data sets into account either directly through the likelihood function or through the prior. Thereby, the computational and logical effort of the fitting remains manageable because the true complexity is hidden.

Here, we demonstrated the joint inference procedure with a specific application from perceptual psychology. Several data sets were requested to be explained by the same model class with the supposition that one parameter is equivalent in all data sets. The procedure can not only be applied in similar cases, but could also be used in the case of other modular models.

For more complex models joint inference could also be applicable, if the complex model can be divided into simpler, overlapping modules which can be tested separately. The parameters that are common to all modules are the shared parameters. It is not necessary that all the modules are described by the same sub-models as was the case in the presented example, but each sub-model has to be estimated in through Bayesian methods for joint inference to be applicable. To take another example from psychophysics, we could have two data sets in which one measured the probability of correct responses similar to our examples above, while the other one measured the time that it took the observer to respond to the stimulus as quickly as possible. The models for response accuracy and reaction time will be quite different overall. The model in equation (8) is very common for response accuracy, while there are many models in the literature for reaction time, e.g. [19]–[21]. In both cases, the dependent variable depends on a parameter that quantifies the visibility of the stimulus: We expect responses to be more accurate for a highly visible stimulus, and we also expect responses to be faster for a highly visible stimulus. For example, drift-diffusion models for reaction time (e.g. [20]) describe descision making as a diffusion process with drift. In these models, the drift (often called 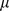) would be a visibility parameter. This parameter is analogous to 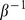 in equation (8) and could be assigned the joint prior distribution presented here.

Furthermore, joint inference can be used to test if two or more data sets can be combined into a single data set, for example the results from several observers in psychology. Here, all model parameters would be treated as shared parameters and the model selection routine returns a criterion for the feasibility of the data set combination.

In general, the problem that we addressed here, is not a new problem. Other methods are available to deal with data collected across multiple conditions, most notably analysis of covariance (ANCOVA) and hierarchical linear regression models. Both methods are based on the assumption that the dependent variable is normally distributed with equal variance/regression slope across conditions. For many real world data sets, normality is valid only in the limit of infinitely large data sets, equality of variance is met only locally, and equality of regression slopes is not met. It is possible to correct the results of ANCOVA for violations of these assumptions and generalized linear mixed models provide a way to extend hierarchical linear models to incorporate non-linear link functions (see [22] for a review). Yet, the ANCOVA is limited to linear dependencies, while generalized linear mixed models are technically much more involved and do not lend themselves to detailed and intuitive assessments of the model's goodness of fit. This is where joint bayesian analysis is helpful. By keeping the structure of each model separate, we can use arbitrarily complex models for individual conditions and integrate information across conditions only through the joint prior. Another and more important difference is the possibility that the different submodels do not need to be structurally equal. This has been elaborated in more detail above.

The general benefits we foresee from joint inference being applied, is that the computational overhead is low and that standard procedures for Bayesian inference can be adopted. Furthermore, the procedure allows a direct model comparison between the joint and isolated models to test the assumption of parameter equality. Here, we used a non-frequentist model selection criterion based on Bayes factors which are readily interpretable.

## Supporting Information

Methods S1 **Determining model posteriors.**
(PDF)
