# Supplementary material for: Joint Bayesian Inference Reveals Model Properties Shared between Multiple Experimental Conditions
Source: PLoS One. 2014 Apr 7;9(4):e91710. doi: 10.1371/journal.pone.0091710 (PMC3977831; doi:10.1371/journal.pone.0091710)
Supplement: Methods S1 — Determining model posteriors. (PDF) [file pone.0091710.s001.pdf]

Appendix S1 Determining model posteriors

Dold & Fründ

Joint Bayesian inference reveals model properties shared between multiple experimental conditions

We used model posterior probabilities to determine whether to prefer a joint fit or the two isolated fits. To do so, we considered a "meta model" with an additional parameter $\mathcal{M}$ that indicated whether the data were generated from the joint model $\mathcal{M} = 1$ or the isolated models $\mathcal{M} = 2$. After having generated samples from the isolated models and from the joint model, we can determine the marginal distribution of $\mathcal{M}$ using Gibbs sampling [1, 2].

Denote the N samples of all parameters from the models estimated with the joint procedure by $\{\theta_i^{(1)}\}_{i=1}^N$, and the samples of parameters from the isolated models by $\{\theta_i^{(2)}\}_{i=1}^N$. To perform Gibbs sampling in the meta model with parameters $\{\mathcal{M}, \theta\}$, we need to consider the full conditional distributions

$$f(\theta|\mathcal{M}, \mathcal{D}), \tag{1}$$

$$f(\mathcal{M}|\theta, \mathcal{D}) = (f(\mathcal{M} = 1|\theta^{(1)}, \mathcal{D}), f(\mathcal{M} = 2|\theta^{(2)}, \mathcal{D})) = (p_1, p_2), \tag{2}$$

where $\mathcal{D}$ denotes the total set of data that have been collected. Once we have determined posterior samples for the models under consideration, we can generate samples from equation (1) by resampling from $\{\theta_i^{(k)}\}_{i=1}^N$. In addition, the parameter p_1 and p_2 in equation (2) are given by

$$p_k \propto \mathcal{P}(\mathcal{D}|\theta^{(k)}, \mathcal{M} = k)\mathcal{P}(\theta^{(k)}|\mathcal{M} = k).$$

With the additional requirement that $p_1 + p_2 = 1$, these two can easily be determined for any parameter sample $\theta_*^{(k)}$.

By alternating between equations (1) and (2), we can generate a Markov chain in the parameter space of the meta model. The stationary distribution of the Markov chain will approximate the posterior distribution of the meta model.

We can further derive an analytical expression for the marginal distribution over $\mathcal{M}$. To this, we consider the transition probabilities

$$\mathcal{P}(\mathcal{M}_k \to \mathcal{M}_\ell) = \int \mathcal{P}(\mathcal{M}_\ell|\theta)\mathcal{P}(\theta|\mathcal{M}_k)\,\mathrm{d}\theta \approx \sum_i \frac{g_\ell(\theta_i^{(k)})}{\sum_j g_j(\theta_i^{(k)})},$$

where g_j is the unnormalised posterior of model j. Denote the transition matrix with entries $T_{k\ell} = \mathcal{P}(\mathcal{M}_k \to \mathcal{M}_\ell)$ by $\mathbf{T}$, then the stationary distribution π should satisfy

$$\pi\mathbf{T} = \pi,$$

with

$$\pi_1 + \pi_2 = 1.$$

Thus for a 2×2 transition matrix, we have to solve the following linear equation system

$$\pi_1 T_{11} + \pi_2 T_{12} = \pi_1 \tag{3}$$
$$\pi_1 T_{21} + \pi_2 T_{22} = \pi_2 \tag{4}$$
$$\pi_1 + \pi_2 = 1. \tag{5}$$

Using the first and the last of these equations, we can see that

$$\pi_2 = \frac{1 - T_{11}}{1 + T_{12} - T_{11}}, \quad \pi_1 = 1 - \pi_2 = \frac{T_{22}}{1 + T_{12} - T_{11}}.$$

References

[1] Casella G, George EI (1992) Explaining the gibbs sampler. The American Statistician 46: 167-174.

[2] Gelman A, Carlin JB, Stern HS, Rubin DB (2003) Bayesian Data Analysis. London: Chapman & Hall/CRC, 2 edition.
